# Plasma Metabolomics Reveals β-Glucan Improves Muscle Strength and Exercise Capacity in Athletes

**DOI:** 10.3390/metabo12100988

**Published:** 2022-10-18

**Authors:** Ruwen Wang, Xianmin Wu, Kaiqing Lin, Shanshan Guo, Yuning Hou, Renyan Ma, Qirong Wang, Ru Wang

**Affiliations:** 1 School of Exercise and Health, Shanghai Frontiers Science Research Base of Exercise and Metabolic Health, Shanghai University of Sport, Shanghai 200438, China; 2 State Key Laboratory of Genetic Engineering, School of Life Sciences, Fudan University, Shanghai 200438, China; 3 National Institute of Sports Medicine, National Testing & Research Center of Sports Nutrition, Beijing 100029, China

**Keywords:** β-glucan, exercise, muscle strength, muscle mass, VO_2max_

## Abstract

The present study aimed to assess the changes in muscle strength and plasma metabolites in athletes with β-glucan supplementation. A total of 29 athletes who met the inclusion criteria were recruited for this study (ChiCTR2200058091) and were randomly divided into a placebo group (*n* = 14) and β-glucan group (*n* = 15). During the trial, the experimental group received β-glucan supplementation (2 g/d β-glucan) for 4 weeks and the control group received an equal dose of placebo supplementation (0 g/d β-glucan), with both groups maintaining their regular diet and exercise habits during the trial. The athletes’ exercise performance, muscle strength, and plasma metabolome changes were analyzed after 4 weeks of β-glucan supplementation. The results showed a significant increase in mean grip strength (kg), right hand grip strength (kg), left triceps strength (kg), and upper limb muscle mass (kg) in the experimental group after the 4-week intervention compared to the preintervention period (*p* < 0.05). A comparison of the difference between the two groups after the intervention showed that there were significant differences between the control group and the experimental group in mean grip strength (kg) and right-hand grip strength (kg) (*p* < 0.05). Athletes in the experimental group showed significant improvements in 1 min double rocking jump (pcs), VO_2max_ (ml/kg-min) (*p* < 0.05). The β-glucan intake increased the creatine-related pathway metabolites in plasma. Overall, these results suggest that 4 weeks of β-glucan supplementation can improve muscle strength in athletes, with the potential to increase aerobic endurance and enhance immune function, possibly by affecting creatine-related pathways.

## 1. Introduction

Muscle strength and quality are key factors to maintain the athletic performance of athletes [1,2]. Nutritional supplementation, mainly including whey protein, creatine, glutamine, l-carnitine, and other nutritional supplements [3,4], is a pivotal means to maintain and enhance muscle function. At the same time, more and more research evidence has shown that natural molecular products play a regulatory role in the process of myocyte proliferation and differentiation [5]. In 2021, Frampton J et al. [6] analyzed the relationship between natural product (dietary fiber) intake and body composition, glucose homeostasis, and skeletal muscle strength. They found that higher dietary fiber intake was significantly associated with higher total lean mass, limb lean mass, and grip strength. It was significantly associated with lower body weight, BMI, total fat mass, trunk fat mass, fasting blood glucose, fasting insulin, and insulin resistance. Therefore, appropriate dietary fiber supplementation may promote the maintenance of muscle function.

As one of the most common natural soluble dietary fibers, β-glucan is mostly derived from plants, such as oats, barley, and seaweed [7]. Many studies have reported the biological functions of β-glucan, such as regulating the immune system, preventing tumors, and reducing the absorption of cholesterol and fat [8,9,10]. Brain dysfunction may have profound effects on metabolic and other physiological functions [11]. β-glucan attenuates cognitive impairment via the gut–brain axis in diet-induced obese mice [12]. Previous studies have shown that the regular intake of dietary fiber such as β-glucan has health benefits such as lowering blood glucose, improving blood lipids, affecting gut microbiota, and enhancing immune function [13,14,15,16].

Hannah A. Zabriskie studied the effects of yeast β-glucan supplementation on exercise-induced muscle injury and inflammation [17]. A significant increase was observed in serum myoglobin in the yeast β-glucan group compared with baseline [18]. Meanwhile, Chao Xu et al. [18] investigated the effect of oat β-glucan on muscle function in rats, suggesting that oat β-glucan can reduce cell damage in liver and muscle tissue. These findings suggest that β-glucan may have a positive effect on muscle tissue. At the same time, some studies have shown that β-glucan can reduce fatigue recovery time [19] and improve endurance exercise performance in rats [18,20]. Yan Li et al. [21] analyzed the effects of β-glucan on myocyte metabolism and antifibrosis in cell experiments, and found that myotube cells treated with β-glucan showed a transition trend from fast muscle fibers to slow muscle fibers, and the oxidative metabolism of myocytes was improved.

In conclusion, the existing studies suggested that β-glucan may be a nutritional supplement that achieves comprehensive health effects and has the potential to improve skeletal muscle function. However, the mechanism of how β-glucan improves muscle strength and exercise performance has not been studied. Therefore, in this study, athletes were selected as subjects to explore the possible mechanism of β-glucan supplementation on muscle strength through plasma metabolomics.

## 2. Material and Methods

### 2.1. Participants

The subjects in this study were recruited from June 2021 to July 2021 from Shanghai University of Sport. A total of 29 professional table tennis players were recruited through recruitment advertisements and offline publicity. (Considering the possible influence of different sports on the results, athletes from the same specialty were recruited.) General information was collected before the participants were enrolled. The study was approved by the Ethics Committee of the Institute of Sports Medicine of the General Administration of Sport of China (approval no.: 202109). International Clinical Registration Number: ChiCTR2200058091. All eligible subjects signed the informed consent form before enrollment.

The sample size of this study was calculated based on previous studies [22], with the test power (1-β) set to 0.80, the test level (α) set to 0.05, and the effect size (POWER) set to 1.05. After calculation, the total sample size was 24 cases, with 12 cases in each group. Considering the loss to follow-up, 15% loss to follow-up was estimated. Therefore, a total sample size of 28 subjects was required in the actual study, and a minimum of 14 subjects were included in each group.

### 2.2. Inclusion of Study Subjects

(1) Inclusion criteria
  ① Professional table tennis players with a national certificate of Level 2 and above.
  ② Age: 18–23 years old.
  ③ Body mass index: 18.5–23.9 kg/m^2^.
  ④ Consent and sign the informed consent form.
(2) Exclusion criteria
  ① Have cardiovascular, liver, kidney, respiratory diseases, and any chronic or malignant diseases.
  ② Allergies or intolerances.
  ③ Participate in other nutrition studies.

### 2.3. Interventions

#### 2.3.1. Randomization

**Figure 1:**
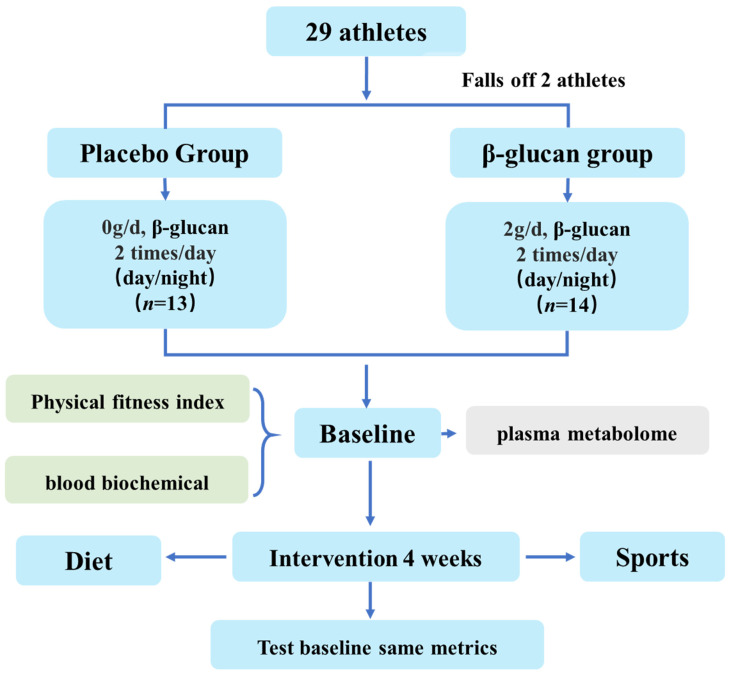
The trial flow.

This study was a single-center, single-blind, randomized controlled trial of table tennis players. All eligible subjects were randomly divided into two groups in a 1:1 ratio: placebo group (*n* = 14) and β-glucan group (*n* = 15). The randomization table was generated by SPSS statistical software. The randomization was performed by people not related to the intervention trial, and the randomization table was kept by the principal investigator. The random numbers were hidden in an opaque envelope that could be opened only after the subjects completed their baseline assessment. The trial flow was shown in Figure 1.

#### 2.3.2. Intervention Protocol

(1) Health lectures

Prior to the intervention, all subjects received two health lectures (once per hour) to ensure that subjects were familiar with the entire study process. First of all, the first class was introduced to the process of the study and how to maintain a healthy lifestyle in daily life (e.g., a balanced and nutritious diet, adequate and good sleep, appropriate physical exercise). In the second class, subjects learned how to use a food diary to record food, to weigh food on a digital scale, and to wear an accelerometer.

(2) Nutritional supplement program

Twenty-nine subjects were randomly divided into two groups: the control group (*n* = 14) and the experimental group (*n* = 15). Subjects in both groups were advised to maintain their normal eating habits and were not allowed to take any additional dietary supplements. During the study period, the β-glucan supplement (2 g/d β-glucan) and placebo supplement (0 g/d β-glucan) were provided by the Institute of Sports Medicine of the General Administration of Sport of China and did not contain any components which would impair human function. The experimental group mainly contained 0.4 g/100 mL of β-glucan, while the placebo group contained the same ingredients except for the absence of β-glucan. Two supplements (250 mL twice a day) were provided by two staff members in the same opaque container before breakfast (7:30 a.m.) and dinner (6:00 p.m.) for 4 weeks. Supplemental doses were given as described in previous studies [23,24,25].

(3) Dietary records

To control diet and avoid large differences in total energy expenditure during the study period, all subjects were advised to maintain their habitual dietary intake and eat in the school cafeteria. In addition, subjects were also asked to record all beverages or foods consumed (type of food, composition, volume or weight consumed, etc.) on three consecutive days per week (including at least one weekend) during the study period. The study staff used nutrition software (the Menthol Dietitian APP) to analyze the total calories during the study period.

(4) Exercise records

In this study, the physical activity level of table tennis players during the trial was assessed by wearing an accelerometer (ActiGraph wGT3X+, bandetek, Pensacola, FL, USA). Participants were asked to wear the accelerometer to the left side of their waist for 7 consecutive days, removing it only when they went to bed, took a shower, or performed swimming exercises. At the end of the wear, Actilife (Version 6.11.9, https://www.actigraph.nl/en/product/11/actilife-6.html) (accessed on 5 May 2022) was used to process and analyze the recorded data, and check whether the test data met the criteria: wear time ≥ 480 min per day, wear time ≥ 4 days (at least one day was a weekend). If the data were found to be unqualified after data analysis, the subjects would be required to wear the test data again. The physical activity measurement parameters were set according to the previous study [26]. Light physical activity: 200 bpm–2689 BPM. Moderate physical activity (2690 to 6166) and high intensity physical activity (≥6167).

### 2.4. Test Indicators

#### 2.4.1. Primary Outcome Measures

(1) Muscle strength [27]

The Portable Physical Function Assessment and Testing System (Kinvent Monpellier, KINVENT, Montpellier, France) was used [27]. The test items included grip strength, triangle strength, biceps strength, triceps strength, and quadriceps strength. The test items included: grip strength, triangle strength, triceps strength, and quadriceps strength. All muscle strength tests were completed by the same tester.

  ① Grip strength

Subjects were asked to sit and stand with elbow flexion at 90°, upper arm against the side of the torso, holding a grip gauge. The grip strength time was 5 s, the interval was 10 s, the right hand first 3 times, the left hand after 3 times, and the left hand was taken as the average of the 3 tests. Mean grip strength = (mean left hand grip strength + mean right hand grip strength)/2, mean grip strength index = mean grip strength/body weight.

  ② Deltoid strength

Subjects were required to sit and stand with fists in hand, fist center facing inward, straight arm, and shoulder abduction at 45°. The instrument was placed above the elbow joint. Subjects were instructed to exert force on shoulder abduction to make muscle isometric contraction. The duration was 5 s/time, the interval was 10 s/time, 3 times on the right side, 3 times on the left side, and the triangle muscle strength on the left and right sides were taken as the average value of the 3 tests. Average deltoid strength = (average left deltoid strength + average right deltoid strength)/2.

  ③ Biceps strength of brachii

The subjects were asked to sit and stand, make a fist, bend the elbow 90°, the upper arm closed to the torso side, and the instrument was placed above the distal forearm, as well as asked to bend the elbow hard so that the muscles performed an isometric contraction, while paying attention that the test subjects did not shrug while keeping the upper body motionless. The duration was 5 s, and the interval was 10 s, 3 times on the right hand, and 3 times on the left hand. Mean biceps strength = (mean left biceps strength + mean right biceps strength)/2.

  ④ Triceps muscle strength

The subjects were asked to sit and stand, make a fist, bend the elbow 90 degrees, the upper arm closed to the torso side, while the instrument was placed under the distal forearm, and they were asked to stretch the elbow hard so that the muscles performed an isometric contraction. The experimenter paid attention so that the test subjects did not shrug while keeping the upper body still. The duration was 5 s, the interval was 10 s, the right hand 3 times, the left hand 3 times. Mean triceps strength = (mean left triceps strength + mean right triceps strength)/2.

  ⑤ Quadriceps muscle strength

Subjects were asked to sit and stand with legs suspended in the air, knees extended 60°, thighs close to the seat, while the instrument was placed in front of the distal tibia, and the patients were asked to stretch their legs to make an isometric muscle contraction. The duration was 5 s and the interval was 10 s, 3 times on the right side, and 3 times on the left side. Mean quadriceps strength = (mean quadriceps strength on the left side + mean quadriceps strength on the right side)/2.

(2) Muscle mass

All subjects were tested for muscle content using a whole-body dual-energy X-ray absorptiometry scanner (GE Prodigy Lunar DXA, Prodigy, White Plains, NY, USA) [28], including total lean mass, upper limb lean mass, lower limb lean mass, trunk lean mass, Android lean mass, and Gynoid lean mass. Prior to the scan, the subjects were instructed not to wear any clothing or accessories with metal zippers or buttons. During the scan, the subject lied flat in the DXA scan area. Then, the tester clicked into the computer test interface, entering the height, weight, date of birth, and gender of the subject, and started the scan. The test time was about 10 min. All calibration and scanning of the whole-body dual-energy X-ray absorptiometry scanner was performed by the same investigator.

#### 2.4.2. Secondary Outcome Measures

(1) Height and weight

The height (accurate to 0.01 cm) and weight (accurate to 0.01 kg) of each subject were measured using the Human body tester of Shanghai Institute of Physical Education. The subjects were required to remove their shoes, socks, and heavy clothing before the measurement.

(2) Blood pressure

The systolic blood pressure (mmHg) and diastolic blood pressure (mmHg) of each subject were measured by the blood pressure pulse tester of Shanghai Institute of Physical Education. The subjects were asked to extend their right upper arm into the cuff sleeve in a quiet state and wait for the measurement result. To ensure the accuracy of the measurement, the blood pressure was measured three times with a 1 min rest in between, and the average of the three measurements was calculated.

(3) Body composition [28]

The measurement method of fat content in this study was the same as that of muscle content. The whole-body dual-energy X-ray absorptiometry scanner was used to measure the fat content. The specific indexes included body fat percentage, total fat content, upper limb fat content, lower limb fat content, trunk fat content, Android fat content, and Gynoid fat content.

(4) Aerobic endurance
  ① 1 min double shake jump

The 1 min double shake jump was an index reflecting the special endurance quality of the table tennis players [29]. Before the test, each subject would adjust the size of the rope to the level of his chest [30]. When the test began in earnest, the researchers would record the number of times the subjects double shake as fast as they can in one minute. If the rope stopped during the test when it got stuck in the subjects’ legs, the participants were immediately verbally encouraged to continue jumping.

  ② Maximal oxygen consumption (VO_2max_)

The VO_2max_ exercise program adopted the Bruce running program. Before the test, subjects wore a metabolic system (COSMED K5, COSMED, Albano Laziale, Italy) and a heart rate sensor (POLAR H10, Polar, NY, USA) and then started the test on a treadmill (Pulsar, H/P/Cosmos, pulsar, Rhede, Germany). For the tests, the researchers used the Borg scale every 3 min to ask participants their self-fatigue level (Rating of Perceived Exertion, RPE). Subjects could stop the test (i.e., considered to be consuming the most oxygen) if they met two of the following three criteria: A. Run to exhaustion or voluntarily determined; B. Respiratory exchange ratio above 1.10; C. Maximum heart rate was reached (maximum heart rate = 220—age).

(5) Immune function

Blood samples would be collected from 7:30 to 8:30 a.m. at Shanghai University of Sport. The subjects were required to keep an empty stomach for 12 h, which meant they were forbidden to eat or drink after 20:00 the night before the blood collection. The volume of blood collected from each subject was 5 mL. The blood sample would be centrifuged at 3500 R/min for 15 min. The obtained plasma samples were stored in a cooler at −80 °C and tested by Golden Domain Medical Testing. The analysis included: interleukin-6 (IL-6), tumor necrosis factor-α (TNF-α), immunoglobulin M (Immunoglobulin M, IgM), C-reactive protein (CRP).

(6) Blood lipids test

In this study, four measurements of blood lipid were the same as immune indicators, and the analysis included: triglyceride (TG), cholesterol (CHOL), high-density lipoprotein cholesterol (HDL-cholesterol), HDL-C, and low-density lipoprotein cholesterol (LDL-C).

### 2.5. Sample Preparation and LC-MS/MS Analysis

The SCIEXSCIEX QTRAP 6500+ mass spectrometer and a Shimadzu LC30AD liquid chromatography system was used to analyze the supernatant. The Waters XBridge Amide (100 mm × 4.6 mm i.d., 3.5 μm) was used for LC separation. A 5μL sample was needed. The electrospray ionization mass spectra were acquired in positive ion mode (4850 V ion spray voltage) and negative ion mode (4500 V ion spray voltage), respectively. The multiple reaction monitoring (MRM) acquisition methods were used to collect MS information simultaneously. The heated capillary temperature was maintained at 475 °C. The curtain gas flow, nebulizer, and heater gas were set to 25, 33, and 33 arbitrary units, respectively.

### 2.6. Statistical Analysis

The Shapiro–Wilk test was used to evaluate the normality of each index, and the Levene test was used to verify the homogeneity of variance. Paired *t*-test or Wilcoxon sign rank test was used for intragroup differences and the independent sample *t*-test or Wilcoxon sign rank test was used for intergroup differences according to the normal distribution and homogeneity of variance of each index, while *p* < 0.05 was set as the level of significance.

Data were analyzed using R (version 4.2; https://www.r-project.org (accessed on 5 May 2022)) software. Multivariate statistical analyses were applied to discriminate individuals with or without β-glucan supply using the adonis2 function in R (permutations = 10,000). The principal component analysis (PCA) was carried out based on the Bray–Curtis dissimilarity matrix, with significance levels determined by the PERMANOVA test. PERMANOVA [31] was conducted on the plasma metabolite profiles of the samples to assess the effect of each clinical measure using the Bray–Curtis dissimilarity and 999 permutations in R (3.5.0, vegan package) [31]. Clinical measures with *p* < 0.05 were considered salient to associate with plasma metabolites. The biological pathways of key metabolites that manifested significant differences between groups were annotated. Biological pathway analysis was performed through metabolite set enrichment analysis using the MetaboAnalyst tool suite under the Kyoto Encyclopedia of Genes and Genomes (KEGG) database [32]. Nonparametric two-tailed Wilcoxon rank sum tests were used throughout the study for statistical testing between groups. Correlations between clinical variables and plasma metabolites were assessed by Spearman’s rank correlation coefficient. Significance was defined as *p* < 0.05 and a trend was defined as 0.05 < *p* < 0.1. Data are presented as a mean ± SD.

## 3. Results

A total of 29 table tennis professional athletes were firstly recruited for a basic information questionnaire and test indicators. They were divided randomly into two groups: 14 athletes for the control group and 15 athletes for the β-glucan supplement group. During the process of the experiment, two people quit, and thus a total of 13 people in the control group and 14 people for the experimental group were finally included for further statistical analysis.

As shown in Table 1, the average age of the control group was 19.38 ± 0.96 (years old), the average height was 170.45 ± 8.98 (cm), the average weight was 67.44 ± 12.64 (kg), the average systolic blood pressure was 124.46 ± 15.59 (mmHg), and the average diastolic blood pressure was 70.46 ± 8.39 (mmHg). The average exercise years were 12.85 ± 1.34 (years). The average age of the experimental group was 19.79 ± 0.89 (years), the average height was 173.29 ± 8.62 (cm), the average weight was 66.6 ± 7.16 (kg), the average systolic blood pressure was 120.71 ± 11.59 (mmHg), the average diastolic blood pressure was 71.86 ± 12.18 (mmHg), and the average exercise time was 12.34 (mmHg). The average exercise years were 12.14 ± 2.14 (years). There was no significant difference in general data between the two groups. The food diary was used to record the diet of the two groups. The results showed (Table 1) that there were no significant differences in average total caloric intake (kJ), average protein intake (G), average fat intake (G), and average carbohydrate intake (G) between the two groups during the whole intervention. Exercise was recorded in both groups by wearing accelerometers. The results showed (Table 1) that there were no significant differences in total physical activity (min/day), light physical activity (min/day), moderate physical activity (min/day), and high physical activity (min/day) between the two groups during the whole intervention (*p* > 0.05). These results indicated that the physical activity status of the two groups was consistent throughout the intervention.

### 3.1. Effects of β-Glucan on Muscle Strength in Athletes

As shown in Table 2, after 4 weeks of intervention, the average grip strength of the experimental group was significantly increased from 29.64 ± 7.63 (kg) to 30.99 ± 6.96 (kg) (*p* < 0.001), and the right-hand grip strength was significantly increased from 30.45 ± 6.83 (kg) to 32.85 ± 5.78 (kg) (*p* < 0.001). The left triceps muscle strength was significantly increased from 18.74 ± 6.15 (kg) to 21.05 ± 6.1 (kg) (*p* < 0.05). However, in the control group, we did not observe any differences in these parameters. The difference between the two groups before and after intervention showed that the average grip strength (kg) and right-hand grip strength (kg) were significantly increased (*p* < 0.01) only in the experimental group.

### 3.2. Effects of β-Glucan Supplementation on Aerobic Endurance of Athletes

As shown in Table 3, in the experimental group, the lean body mass of the upper limb was significantly increased from 5.58 ± 1.47 (kg) to 5.60 ± 1.41 (kg) (*p* < 0.05), while the other muscle content indexes had no significant difference (*p* > 0.05). The 1 min double swing jump was significantly increased from 76.86 ± 19.37 to 86.36 ± 15.52 (*p* < 0.05). VO_2max_ increased from 41.95 ± 4.83 (ml/kg·min) to 43.62 ± 4.34 (ml/kg min) (*p* < 0.05). After 4 weeks of intervention, the VO_2max_ of the control group increased from 41.03 ± 4.62 (ml/kg·min) to 42.05 ± 4.22 (ml/kg·min), without significant difference.

Other indicator results, as shown in Table 4, show that TNF-α was significantly decreased from 5.52 ± 0.86 (pg/mL) to 4.51 ± 0.73 (pg/mL) in the experimental group (*p* < 0.001), while IL-6, IgM, and CRP were not significantly different.

### 3.3. Plasma Metabolomic Profiling Reveals an Apparent Distinction between β-Glucan Treatment and Placebo Group

**Figure 2:**
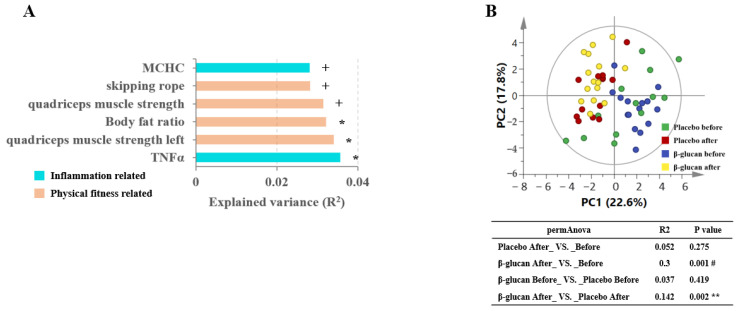
Effects of β-glucan on the metabolite composition of plasma metabolomes in athletes. (**A**) The effect size of phenotype indices contributed significantly to the variance (R2) of the plasma metabolome ( + *p* < 0.1, * *p* < 0.05, ** *p* < 0.01, # *p* < 0.001). (**B**) The principal component analysis (PCA) of the plasma metabolites between the placebo and β-glucan groups based on the permutational multivariate analysis of variance (PERMANOVA test with 10,000 permutations) ( + *p* < 0.1, * *p* < 0.05, ** *p* < 0.01, # *p* < 0.001).

We identified 124 metabolic features (see Methods) across 56 samples. Among them, 57 features (45.97%) were altered in the β-glucan group and 41 features (33.06%) were altered in placebo group (*p* < 0.05). The inflammation-related features and physical-fitness-related features of both groups were significantly associated with the plasma metabolome (Figure 2A, PERMANOVA test, + *p* < 0.1, * *p* < 0.05). Additionally, we found that TNFα and quadriceps muscle strength exerted statistically significant effects on the plasma metabolome of β-glucan group (Figure 2A, PERMANOVA test, * *p* < 0.05). 

However, as shown in Figure 2B, the analysis result shows that R2 is very low, which may be caused by the small sample size of the population. After referring to the relevant literature [31], the analysis continued. Principal component analysis (PCA) of all of the significantly altered metabolic features revealed significant shifts after the β-glucan treatment by comparing with the β-glucan before group (Figure 2B, *p* = 0.001, PERMANOVA test with 10,000 permutations) and the placebo before group (Figure 2B, *p* = 0.002), and no significant changes were observed among the placebo groups (Figure 2B, *p* = 0.275), between the β-glucan before group, and the placebo before group (Figure 2B, *p* = 0.419). 

**Figure 3:**
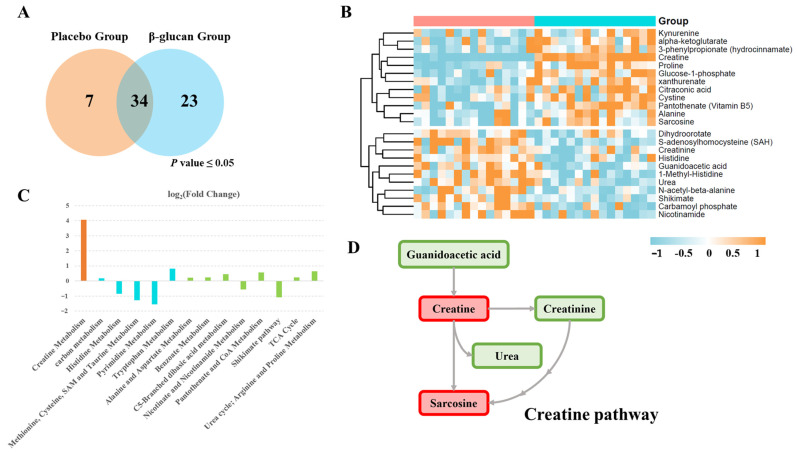
β-glucan supplementation activates the creatine signaling pathway in plasma. (**A**) Venn diagrams resuming the number of plasma metabolites significantly changed (*p* < 0.05) in the placebo group and β-glucan group. (**B**) The heatmap showing changes in the relative content of plasma metabolites after β-glucan treatment. (Wilcoxon rank sum tests;). (**C**) Major metabolic pathways involved in the differentially enriched metabolites after β-glucan treatment (count indicates metabolites enrolled in each pathway). (**D**) Reaction steps for creatine pathway. Metabolites increased after β-glucan treatment are shown in red, whereas the metabolites decreased are shown in green.

Venn diagrams were used to depict the significantly altered (*p* < 0.05) plasma metabolic features in both groups (Figure 3A). Around 28 percent of all significantly altered metabolites were shared between the β-glucan group and placebo group. Twenty-three metabolites were especially changed only after the β-glucan treatment, which indicates that those metabolites may act as direct or indirect targets of β-glucan. Half of the relative content of these 23 metabolites were increased after the β-glucan treatment, while the others were decreased according to the relative content heatmap (Figure 3B). Creatine, which was reported to improve muscle performance in healthy individuals [33], was significantly increased after the β-glucan treatment. Of note, the metabolites engaged in the creatine pathway (Figure 3C) were altered after the β-glucan treatment, including guanidinoacetic acid, sarcosine, urea, and creatinine. Similarly, these 23 metabolites were found in pathways such as the histidine metabolism, methionine metabolism, pyrimidine metabolism, and tryptophan metabolism (Figure 3D). 

**Figure 4:**
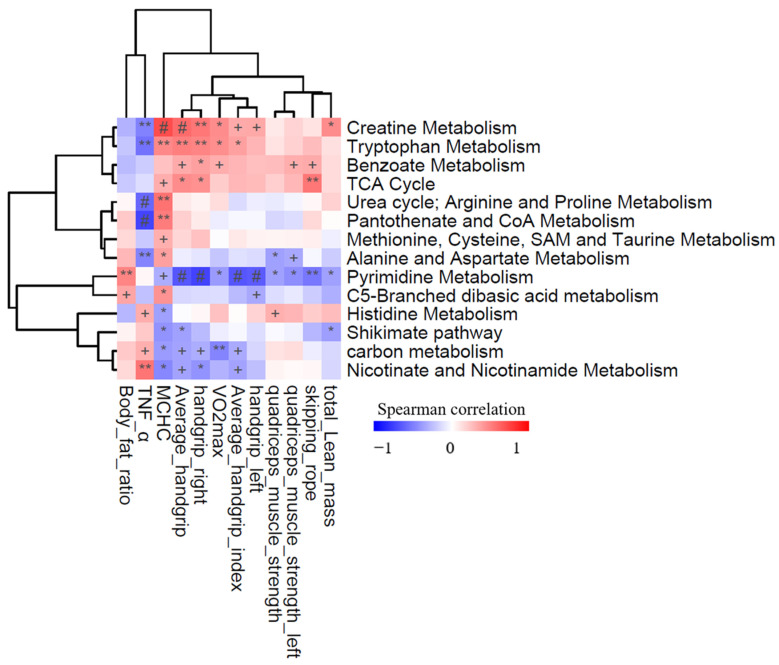
Correlation of β-glucan supplementation with athletic performance. Relationships between phenotypes and the concentration of metabolism pathways. The heatmap displays the Spearman correlation coefficients between phenotypes and metabolism pathways. The significance levels in the correlation tests are denoted as: + *p* < 0.1; * *p* < 0.05; ** *p* < 0.01; # *p* < 0.001. Details of correlation were shown in Supplementary Figure S1.

These metabolic pathways concomitantly changed with the corresponding β-glucan-associated phenotypes (Figure 4). We thus concluded that the alterations of metabolites in the β-glucan intervention group was associated with β-glucan-mediated creatine metabolism, tryptophan metabolism, histidine metabolism, and carbon metabolism. More importantly, the changes of TNF-α were significantly negatively correlated with the content of the creatine metabolism, while the average handgrip and VO_2max_ were significantly positively correlated. These findings suggest that metabolites involved in the creatine metabolism may be potential diagnostic biomarkers of β-glucan treatment.

## 4. Discussion

Muscle strength is recognized as a key factor in maintaining athletic performance. At the same time, lean mass is the main component of skeletal muscle tissue and also an important part of maintaining human life and health. The loss of lean mass will directly affect the health status or athletic performance of athletes. Therefore, it is of great significance to optimize the strength and quality of skeletal muscle for athletes. 

Most studies believed that the metabolic absorption of β-glucan in vivo was mainly through the action of different bioflora in the large intestine to decompose it into SCFAs, such as acetic acid [34], propionic acid, and n-butyric acid, and then transfer it into the body to play a role. β-glucan could also directly bind to immune cell receptors in vivo due to its special helical structure, thus playing an immunomodulatory role [35]. Hong Feng et al. [36] labeled barley and yeast β-1, 3-glucan with fluorescein and tracked its changes in animals. They found that the labeled β-1, 3-glucan could be taken up by macrophages, which could transport them to the blood, spleen, lymph nodes, and bone marrow. These studies suggested that β-1, 3-glucan, as a low-molecular-weight polysaccharide, could enter the body in a special form and play a role. In this study, the muscle glycogen content of oat β-glucan was measured before and after intervention, and it was found that the muscle glycogen of the rats after β-glucan intervention was significantly increased. β-glucan intake has positive effects on glycemic control and insulin sensitivity in diabetic patients, but the mechanism is still unclear. In the future, more novel ways (e.g., single-cell transcriptomics) can be used to explore the role of β-glucan in the transition of β-cells between different states [37,38].

Hannah A. Zabriskie et al. [17] found that β-glucan significantly increased myoglobin in healthy adults. Muscle glycogen and myoglobin are energy and oxygen storage substances distributed in the sarcoplasm, and their increased content helps the body to provide energy and oxygen supply during muscle contraction, thereby increasing muscle strength. In addition, Yan Li et al. [21] analyzed the effects of β-glucan on myocyte metabolism and antifibrosis in cell experiments and found that myotube cells treated with β-glucan showed a transition from fast to slow muscle fibers, and the expression levels of slow muscle-fiber-related markers were significantly increased. There was a close relationship between muscle glycogen and muscle fiber type. Studies have shown that increasing the number of slow muscle fibers can significantly increase the content of muscle glycogen, thereby increasing muscle strength [39]. These studies suggested that the improvement of muscle strength by β-glucan may be related to muscle glycogen, myoglobin, and muscle fiber type increase, but the specific mechanism needed to be further studied.

As the health benefits of β-glucan have gained public recognition, some researchers have begun to explore the effects of β-glucan on exercise performance [18]. In an animal study, Heeok Hong found that β-glucan could significantly increase the running exhaustion time of rats, which may be explained by the mechanism that β-glucan alleviates fatigue by inhibiting c-fos and c-Jun, which are early onset genes involved in cell growth, differentiation, and development in the brain of tired rats, and thereby improving the exercise ability of rats [20]. Chao Xu et al. [19] found that β-glucan supplementation significantly increased the exhaustion exercise time and liver glycogen concentration in rats, suggesting that β-glucan might enhance exercise endurance by increasing the storage of liver glycogen. In addition to the muscle-related primary outcome measures, we measured the 1 min double swing rope jump, which reflected the specific endurance quality of table tennis players, and the classical indicator of aerobic endurance, VO_2max_. The results showed that the 1 min double-shake rope skipping and VO_2max_ of the experimental group were significantly improved after 4 weeks of intervention compared with those before intervention (*p* < 0.05), which was consistent with the results of previous animal studies. This study further proved that β-glucan can promote the aerobic capacity of the body in humans. Different studies revealed that the mechanism of β-glucan on improving aerobic endurance of the body is not consistent, so further exploration and research were needed. However, there were few studies on the effect of β-glucan on the aerobic capacity of the body, which was still in the exploratory stage. Our research provided a supporting theoretical basis for filling this part of the content and provided ideas for further research on related mechanisms in the future.

It has been found that dietary β-glucan changed the levels of blood urea nitrogen, lactic acid, and creatine, and alleviated body fatigue [19]. The results also suggested that creatine supplementation before, during, and after resistance training was an effective intake strategy for improving muscle strength [40]. Exercise further promotes the body’s metabolic process, improves lipid metabolism, and increases energy consumption [41,42]. In several meta-analyses and review articles related to creatine supplementation, creatine supplementation was found to be readily and positively associated with muscle endurance and exercise performance [43,44,45,46,47]. It was also possible that creatine supplements increase calcium reuptake by the sarcoplasmic reticulum, which would lead to faster actin-myosin cross-bridge cycling during repeated muscle contractions [48]. Overall, these results suggested improved muscle strength and endurance.

Also of interest are the beneficial effects of supplementation with foods containing oat β-glucan on carbohydrate metabolism and blood pressure in obese subjects, as shown in the results of a randomized, double-blind, controlled clinical trial [49]. Studies have shown that β-glucan supplementation in hypertensive subjects has some hypotensive effect [50]. Animal studies have also confirmed that β-glucan was a potential antihypertensive agent that can alleviate cardiac dysfunction [51]. It has also been demonstrated that there was a circadian variation in blood pressure in patients with chronic musculoskeletal pain [52], and that the variation in blood pressure also affected muscle function, making β-glucan a potential cost-effective treatment [52]. The current experiment collected data related to blood pressure, and likely due to the small sample size, there was no significant difference between the two groups. In future experiments, the effect of blood pressure will be considered, which will provide a better guidance on when to supplement β-glucan and the effect of circadian rhythm on further muscle performance enhancement.

## 5. Conclusions

In this study, a 4-week β-glucan supplementation improved the muscle strength of athletes, had the potential to improve aerobic endurance, and enhanced the immune function of the athletes, which provided a new idea for the development of a new type of healthy nutritional supplementation for athletes.

## 6. Limitations of Study

There are certain limitations of our study. First, due to the small sample size and short intervention time of the selected study subjects as athletes, this study did not fully reflect the effect of β-glucan supplementation, such as the effect on the lipid metabolism. At the same time, due to the small sample size, the value of R2 was small when the histological test was performed, and the follow-up analysis was still conducted after referring to the relevant literature, which may cause the data to not truly reflect the experimental results and mask some of the confounding factors, leading to the inaccuracy of the prediction, which needs to be further validated by increasing the sample size in the future. Second, the intervention dose selection in this study referred to the dose set in the previous literature study without a gradient test, and in the future requires further improvement of the intervention program and the determination of the optimal supplemental dose for athletes.

## Figures and Tables

**Table 1:** Baseline characteristics of experimental groups.

Index	Placebo Group (*n* = 13)	β-Glucan Group (*n* = 14)	*t*	*p*
Baseline information				
Age (years)	19.38 ± 0.96	19.79 ± 0.89	1.124	0.271
Height (cm)	170.45 ± 8.98	173.29 ± 8.62	0.836	0.411
Body weight (kg)	67.44 ± 12.64	66.6 ± 7.16	−0.214	0.832
Systolic pressure (mmHg)	124.46 ± 15.59	120.71 ± 11.59	−0.712	0.483
Diastolic blood pressure (mmHg)	70.46 ± 8.39	71.86 ± 12.18	0.344	0.734
Years of exercise (year)	12.85 ± 1.34	12.14 ± 2.14	−1.012	0.403
Nutrition				
Total energy (kJ)	8267.78 ± 1877.55	7746.94 ± 1858.54	−0.724	0.476
Protein (g)	90.68 ± 16.07	74.87 ± 23.33	−2.351	0.055
Fat (g)	81.68 ± 21.73	73.99 ± 21.63	−0.921	0.366
Carbohydrate (g)	201.81 ± 72.47	187.77 ± 61.64	−0.543	0.592
Dietary fiber (g)	7.13 ± 3.07	5.84 ± 2.63	−1.17	0.252
Activity				
Total physical activity (min/day)	731.99 ± 77.30	769.96 ± 122.42	0.955	0.349
Light physical activity (min/day)	639.24 ± 78.96	657.52 ± 121.44	0.460	0.650
Moderate physical activity (min/day)	79.70 ± 21.77	87.69 ± 33.06	0.735	0.482
High physical activity (min/day)	8.23 ± 9.56	5.12 ± 7.19	−0.960	0.610

Statistical analyses were processed using R software (version 4.2; https://www.r-project.org (accessed on 5 May 2022)). Continuous variable values are expressed as mean ± standard deviation. The Shapiro–Wilk test was used to evaluate the normality of each index, and the Levene test was used to verify the homogeneity of variance. Paired *t*-test or Wilcoxon sign rank test was used for intragroup differences and independent sample *t*-test or Wilcoxon sign rank test was used for intergroup differences according to the normal distribution and homogeneity of variance of each index.

**Table 2:** β-glucan supplementation improves muscle strength in athletes.

Index	Placebo Group (*n* = 13)				β-Glucan Group (*n* = 14)				Change	
	Before	After	*t*	*p*	Before	After	*t*	*p*	*t*	*p*
Average Grip Strength Index	0.44 ± 0.09	0.43 ± 0.09	−0.890	0.391	0.45 ± 0.11	0.46 ± 0.09	2.133	0.053	2.039	0.052
Average grip strength (kg)	29.49 ± 7.69	28.69 ± 7.85	−1.645	0.126	29.64 ± 7.63	30.99 ± 6.96 **	3.623	0.003	3.547	0.002
Right-hand grip (kg)	31.56 ± 7.77	31.07 ± 8.48	−0.753	0.466	30.45 ± 6.83	32.85 ± 5.78 ***	4.837	<0.001	3.555	0.002
Left-hand grip (kg)	27.42 ± 8.02	26.32 ± 7.72	−1.863	0.087	28.82 ± 8.87	29.14 ± 8.6	0.574	0.576	1.759	0.091
Average deltoid strength (kg)	21.13 ± 7.03	19.72 ± 5.37	−1.348	0.203	21.69 ± 4.98	21.98 ± 5.87	0.338	0.741	1.261	0.219
Right Deltoid Strength (kg)	21.92 ± 7.52	20.67 ± 5.73	−1.165	0.267	21.69 ± 4.98	22.65 ± 5.48	1.379	0.191	0.229	0.821
Left deltoid muscle strength (kg)	20.34 ± 6.94	18.77 ± 5.29	−1.348	0.203	21.04 ± 5.82	21.31 ± 6.68	0.341	0.738	0.932	0.360
Average biceps strength (kg)	25.12 ± 8.33	23.97 ± 7.35	−0.863	0.405	23.43 ± 6.69	23.98 ± 6.15	0.489	0.383	1.124	0.272
Right biceps strength (kg)	27.28 ± 8.8	26.19 ± 8.46	−0.805	0.436	25.11 ± 7.82	25.86 ± 5.8	0.578	0.573	0.174	0.863
Left biceps strength (kg)	22.96 ± 8.48	21.74 ± 7.07	−0.818	0.429	21.74 ± 6.43	22.09 ± 7.72	0.391	0.702	0.517	0.610
Average triceps strength (kg)	21.12 ± 5.31	20.85 ± 5.43	−0.376	0.714	20.41 ± 5.71	21.18 ± 5.38	1.324	0.208	1.133	0.268
Right triceps strength (kg)	21.45 ± 5.52	20.97 ± 5.67	−0.516	0.616	20.41 ± 5.71	21.31 ± 4.95	1.528	0.151	−0.392	0.699
Left triceps strength (kg)	20.78 ± 5.59	20.72 ± 5.43	−0.087	0.932	18.74 ± 6.15	21.05 ± 6.1 *	2.571	0.023	−1.949	0.063
Average Quadriceps Strength (kg)	31.28 ± 6.7	32.81 ± 6.07	1.234	0.241	32.54 ± 5.09	33.23 ± 8.14	0.278	0.786	−0.295	0.771
Right quadriceps strength (kg)	32.7 ± 7.05	34.42 ± 6.33	1.262	0.231	32.54 ± 5.09	34.54 ± 8.78	0.763	0.459	−1.230	0.230
Left quadriceps strength (kg)	29.85 ± 7.33	31.2 ± 6.28	0.781	0.450	31.69 ± 6.75	31.91 ± 7.87	0.093	0.927	−0.526	0.603

Statistical analyses were processed using R software (version 4.2; https://www.r-project.org (accessed on 5 May 2022)). Continuous variable values are expressed as mean ± standard deviation. The Shapiro–Wilk test was used to evaluate the normality of each index, and the Levene test was used to verify the homogeneity of variance. Paired *t*-test or Wilcoxon sign rank test was used for intragroup differences and independent sample *t*-test or Wilcoxon sign rank test was used for intergroup differences according to the normal distribution and homogeneity of variance of each index. Compared with before intervention: * *p* < 0.05, ** *p* < 0.01, *** *p* < 0.001.

**Table 3:** Comparison of the index results of the whole-body muscle content before and after the intervention in the two groups of subjects.

Index	Placebo Group (*n* = 13)				β-Glucan Group (*n* = 14)				Change	
	Before	After	*t*	*p*	Before	After	*t*	*p*	*t*	*p*
Total lean body mass (kg)	49.5 ± 9.59	49.29 ± 10.02	−0.708	0.492	50.26 ± 8.14	50.66 ± 8.58	1.528	0.151	1.540	0.136
Upper body lean body mass (kg)	5.34 ± 1.46	5.36 ± 1.54	0.359	0.726	5.58 ± 1.47	5.60 ± 1.41	2.167	0.049 *	1.846	0.077
Lower extremity lean body mass (kg)	17.2 ± 3.51	17.09 ± 3.73	−0.646	0.531	17.44 ± 3.05	17.56 ± 3.13	0.997	0.337	1.123	0.272
Trunk lean body mass (kg)	23.07 ± 4.21	22.98 ± 4.35	−0.513	0.617	23.46 ± 3.46	23.71 ± 3.86	1.080	0.300	1.155	0.259
Android_lean body mass (kg)	3.03 ± 0.59	3.05 ± 0.66	0.486	0.635	3.14 ± 0.54	3.17 ± 0.59	0.947	0.361	0.272	0.788
Gynoid_lean body mass content (kg)	7.71 ± 1.81	7.64 ± 1.87	−0.917	0.377	7.86 ± 1.39	7.97 ± 1.44	1.675	0.118	1.803	0.083
Body fat percentage (%)	0.22 ± 0.08	0.22 ± 0.08	0.339	0.741	0.20 ± 0.08	0.20 ± 0.08	1.396	0.186	0.594	0.558
Total fat content (kg)	14.69 ± 6.49	14.58 ± 6.55	−0.891	0.391	13.19 ± 5.21	13.17 ± 5.05	2.086	0.057	0.369	0.715
Upper limb fat content (kg)	1.36 ± 0.70	1.35 ± 0.70	−0.337	0.742	1.22 ± 0.60	1.22 ± 0.62	0.107	0.917	0.312	0.758
Lower extremity fat content (kg)	4.66 ± 1.62	4.64 ± 1.72	−0.403	0.694	4.71 ± 1.96	4.72 ± 2.01	0.090	0.929	0.310	0.759
Trunk fat content (kg)	8.08 ± 4.21	8.01 ± 4.11	−0.699	0.498	6.72 ± 2.61	6.84 ± 2.61	1.238	0.238	1.356	0.187
Android_fat content (kg)	1.38 ± 0.72	1.40 ± 0.75	0.878	0.397	1.08 ± 0.37	1.10 ± 0.34	0.632	0.539	0.068	0.947
Gynoid_fat content (kg)	2.81 ± 0.96	2.84 ± 0.99	0.907	0.382	2.82 ± 1.14	2.82 ± 1.13	−0.133	0.896	−0.635	0.531
1 min double shake (pcs)	78.27 ± 19.42	83.63 ± 18.75	1.90	0.087	76.86 ± 19.37	86.36 ± 15.52	2.735	0.017 *	0.427	0.673
VO_2max_ (ml/kg·min)	41.03 ± 4.62	42.05 ± 4.22	1.93	0.082	41.95 ± 4.83	43.62 ± 4.34	2.394	0.038 *	0.728	0.475

Statistical analyses were processed using R software (version 4.2; https://www.r-project.org (accessed on 5 May 2022)). Continuous variable values are expressed as mean ± standard deviation. The Shapiro–Wilk test was used to evaluate the normality of each index, and the Levene test was used to verify the homogeneity of variance. Paired *t*-test or Wilcoxon sign rank test was used for intragroup differences and independent sample *t*-test or Wilcoxon sign rank test was used for intergroup differences according to the normal distribution and homogeneity of variance of each index. Compared with before intervention: * *p* < 0.05 VO_2max_, maximal oxygen consumption.

**Table 4:** Comparison of the results of four indicators of blood lipids before and after the intervention in the two groups of subjects.

Index	Placebo Group (*n* = 13)				β-Glucan Group (*n* = 14)				Change	
	Before	After	*t*	*p*	Before	After	*t*	*p*	*t*	*p*
TG (mmol/L)	0.90 ± 0.41	0.97 ± 0.38	0.660	0.522	0.89 ± 0.26	1.00 ± 0.59	0.740	0.472	0.247	0.807
CHOL (mmol/L)	4.29 ± 0.69	4.24 ± 0.64	−0.509	0.620	4.31 ± 0.49	4.35 ± 0.61	0.266	0.795	0.499	0.622
HDL-C (mmol/L)	1.44 ± 0.29	1.39 ± 0.28	−1.469	0.168	1.67 ± 0.33	1.72 ± 0.37	0.871	0.400	1.418	0.168
LDL-C (mmol/L)	2.61 ± 0.56	2.70 ± 0.61	0.919	0.376	2.41 ± 0.31	2.54 ± 0.46	1.180	0.259	0.273	0.787
IL-6 (pg/mL)	2.16 ± 0.28	2.81 ± 1.29	1.740	0.107	2.54 ± 1.50	2.56 ± 0.87	0.061	0.952	−1.230	0.270
IgM (g/L)	1.25 ± 0.66	1.30 ± 0.71	1.723	0.110	1.39 ± 0.56	1.46 ± 0.61	1.727	0.108	0.213	0.833
TNF-α (pg/mL)	5.61 ± 1.34	5.14 ± 1.00	−1.976	0.072	5.52 ± 0.86	4.51 ± 0.73 ***	−5.576	<0.001	−1.852	0.076
CRP (mg/L)	0.97 ± 0.42	0.64 ± 0.46	−2.164	0.051	1.34 ± 1.11	0.79 ± 1.23	−1.160	0.267	−0.424	0.675

Statistical analyses were processed using R software (version 4.2; https://www.r-project.org (accessed on 5 May 2022)). Continuous variable values are expressed as mean ± standard deviation. The Shapiro–Wilk test was used to evaluate the normality of each index, and the Levene test was used to verify the homogeneity of variance. Paired *t*-test or Wilcoxon sign rank test was used for intragroup differences and independent sample *t*-test or Wilcoxon sign rank test was used for intergroup differences according to the normal distribution and homogeneity of variance of each index. Compared with before intervention: *** *p* < 0.001. TG, triglyceride; CHOL, cholesterol; HDL-C, high-density lipoprotein; LDL-C, low-density lipoprotein; IL-6, interleukin-6; IgM, immunoglobulin M; TNF-α, tumor necrosis factor-α; CRP, C-reactive protein.

## Data Availability

The data presented in this study are available within the article.

## Supplementary Materials

The following supporting information can be downloaded at: https://www.mdpi.com/article/10.3390/metabo12100988/s1, Figure S1: Changes in related metabolites after β-glucan supplementation.
