# Supplementary material for: Plasma Metabolomics Reveals β-Glucan Improves Muscle Strength and Exercise Capacity in Athletes
Source: Metabolites. 2022 Oct 18;12(10):988. doi: 10.3390/metabo12100988 (PMC9607031; doi:10.3390/metabo12100988)
Supplement: Supplementary file 1 [file metabolites-12-00988-s001.zip › metabolites-1959084-supplementary.pdf]

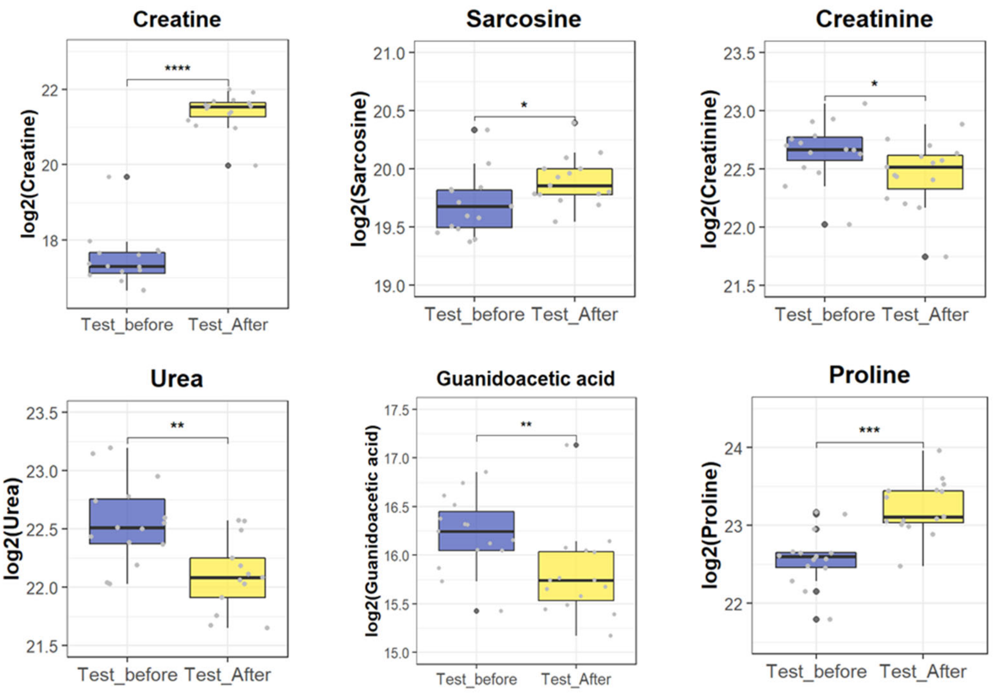

Figure S1. Changes in related metabolites after β-glucan supplementation**.** Wilcoxon rank-sum tests; *$p < 0.05$, **$p < 0.01$, ***$p < 0.001$, .****$p < 0.0001$. Details of correlation were shown in Supplementary Figure1.
